# Comparing potentially avoidable hospitalization rates related to ambulatory care sensitive conditions in Switzerland: the need to refine the definition of health conditions and to adjust for population health status

**DOI:** 10.1186/1472-6963-14-25

**Published:** 2014-01-20

**Authors:** Yves Eggli, Béatrice Desquins, Erol Seker, Patricia Halfon

**Affiliations:** 1 Institute of Health Economics and Management, Centre Hospitalier Universitaire Vaudois and University of Lausanne (Faculty of Business and Economics and Faculty of Biology and Medicine), Route de Chavannes 31, CH-1015, Lausanne, Switzerland; 2 Institute of Social and Preventive Medicine (IUMSP), Centre Hospitalier Universitaire Vaudois, University of Lausanne (Faculty of Biology and Medicine), Route de la Corniche 10, 1010, Lausanne, Switzerland

**Keywords:** Quality assessment, Ambulatory care, Morbidity adjustment, Potentially avoidable hospitalizations

## Abstract

**Background:**

Regional rates of hospitalization for ambulatory care sensitive conditions (ACSC) are used to compare the availability and quality of ambulatory care but the risk adjustment for population health status is often minimal. The objectives of the study was to examine the impact of more extensive risk adjustment on regional comparisons and to investigate the relationship between various area-level factors and the properly adjusted rates.

**Methods:**

Our study is an observational study based on routine data of 2 million anonymous insured in 26 Swiss cantons followed over one or two years. A binomial negative regression was modeled with increasingly detailed information on health status (age and gender only, inpatient diagnoses, outpatient conditions inferred from dispensed drugs and frequency of physician visits). Hospitalizations for ACSC were identified from principal diagnoses detecting 19 conditions, with an updated list of ICD-10 diagnostic codes. Co-morbidities and surgical procedures were used as exclusion criteria to improve the specificity of the detection of potentially avoidable hospitalizations. The impact of the adjustment approaches was measured by changes in the standardized ratios calculated with and without other data besides age and gender.

**Results:**

25% of cases identified by inpatient main diagnoses were removed by applying exclusion criteria. Cantonal ACSC hospitalizations rates varied from to 1.4 to 8.9 per 1,000 insured, per year. Morbidity inferred from diagnoses and drugs dramatically increased the predictive performance, the greatest effect found for conditions linked to an ACSC. More visits were associated with fewer PAH although very high users were at greater risk and subjects who had not consulted at negligible risk. By maximizing health status adjustment, two thirds of the cantons changed their adjusted ratio by more than 10 percent. Cantonal variations remained substantial but unexplained by supply or demand.

**Conclusion:**

Additional adjustment for health status is required when using ACSC to monitor ambulatory care. Drug-inferred morbidities are a promising approach.

## Background

One approach to measuring the quality and availability of primary care is to examine hospital admission rates for conditions that, if treated early and appropriately, are manageable outside the hospital setting. These are known as ambulatory care sensitive conditions (ACSC) [1,2]. Avoiding such hospitalizations represents a substantial gain in terms of costs and patient suffering. Furthermore inpatients’ medical data are available in most countries.

ACSC are identified from the main diagnosis coding of inpatient stays. Several ACSC lists with their associated disease codes (International Classification of Diseases, 9th Revision, Clinical Modification, ICD-9-CM, or International Classification of Diseases 10th Revision, ICD-10) are available [3,4]. The assumption underlying this concept is that timely access to effective primary care should prevent the progression of the condition and thus the need for hospitalization. Depending on the condition, primary care can consist of primary prevention (e.g. for influenza); the early treatment of acute conditions (e.g. for community-acquired bacterial pneumonia, urinary tract infections and gastroenteritis); or the management of chronic diseases (e.g. asthma, congestive heart failure and diabetes).

Potentially avoidable hospitalization (PAH) rates due to ACSC have been found to vary widely across geographical areas and population groups, suggesting that the quality and availability of ambulatory care may be a contributory factor [5-8]. Most conditions generate at least one admission per 10,000 residents, a number considered sufficient to warrant further investigation [9,10]. Globally, PAH conditions account for 8 to 12% of all hospitalizations [9-11], one in five adult non-obstetric Medicare stays, and one in ten among Medicaid beneficiaries [12,13].

PAH have been extensively examined as an indicator of primary care accessibility. In the USA, higher PAH rates have been consistently associated with lower income, African American ethnicity, and lack of insurance coverage, suggesting disparities in primary care access [9,14,15]. The relationship between socioeconomic disadvantage and PAH may also reflect differences in disease prevalence. In comparisons of PAH rates, most studies adjusted only for age and gender [2,14,16]. Some also adjusted for disease prevalence, though this was restricted to a small number of conditions for which such information was available [17-19]. Incorporating self-reported health status and history of chronic conditions significantly weakened the relationship between all socioeconomic variables and PAH [20,21].

Recently, health plan organizations or large physicians’ groups have shown a greater interest in the use of PAH indicators for performance profiling. This represents a major challenge because, in the absence of a proper risk adjustment for patient health status, health plans might improve their performance simply by enrolling healthier individuals. In a recent structured review on possible indicator refinement, clinical experts cited diagnoses from prior hospitalizations within the past year and information derived from pharmaceutical data as important risk adjustment covariates [22].

Other factors for which ambulatory care providers should not be held accountable may also be at work. Variations in PAH rates should not be the result of either differing hospital admission practices or the propensity of the subject to seek medical care [15].

In Switzerland the fact that health insurance is compulsory reduces the problem of access to care. Switzerland is also a highly decentralized federation, with each of its 26 cantons having almost sole responsibility for the organization and financing of their care services [23]. As a result, primary care is organized differently across the country, making it possible to examine the influence of contrasting health care characteristics.

Linking databases of several large insurers’ claims and hospitalization records enabled us to examine PAH by using individual data on a nationally representative cohort of insured persons. Our study aimed to assess the impact of various severity adjustment strategies on the indicator variations by using increasingly detailed information on health status (age and gender only, inpatient diagnoses, outpatient conditions inferred from dispensed drugs data and frequency of care use). We then investigated the association between the properly adjusted indicator and socioeconomic factors and/or care services characteristics.

## Methods

### Studied population

Our study is an observational study based on routine data from four Swiss health insurers for the years 2005 and 2006. Data were collected with the support of the Swiss Federal Office of Public Health [24]. The studied population included 2,022,019 individuals who were insured with one of those Swiss health insurers in 2005. They were followed from January 1 until December 31, 2005 (335,538), and until December 31, 2006 (1,686,481) for those who did not change insurer during the observation period (in Switzerland changes are only permitted at the end of the year). All their health service bills and drugs claims were systematically collected and matched against hospital medical records through an anonymous linkage code established by the Swiss Federal Statistical Office (SFSO) encryption process (only the sequential number of the patient was supplied) [25]. Hospital data supplied by the Federal Statistical Office (inpatient diagnoses) are publicly available. Insurers’ data (dispensed drugs and ambulatory services) are not publicly available and were supplied only for the research project supported by the Federal Public Health Office, with the prerequisite of using the anonymous linkage code procedure of the Federal Statistical Office. All data were anonymous and did not include any information which might identify the individual (date of birth, ZIP code, etc.) [26].

### Outcome

The outcome was the number of PAH occurring between 1 July 2005 and 31 December 2005/2006, with 182 and 547 days of follow-up respectively. An ACSC was retained from the main inpatient diagnosis. Conditions were identified by ICD-10 codes listed by Purdy et al. and established from an exhaustive literature review updated in 2007 [4]. A few additional codes were added for specific conditions (e.g. gastroenteritis due to food poisoning and otitis externa; see Additional file 1 for list). The following diagnoses were removed from Purdy’s list (see Additional file 1 for codes). It is probable that minor dental problems, which rarely require hospitalisation, are linked to admission practices rather than the efficiency of primary care. A few conditions did not fit into the clinical definition of ACSC: oesophagitis and oesophageal reflux (perforated or bleeding ulcer category): and aplastic or auto-immune anaemia (deficiency anaemia category). We also excluded eclampsia (convulsions category) and late complications of diabetes (which signal problems with the care provided at a much earlier stage) because these could not be managed by primary care during the observation period. Cases with a comorbid condition requiring hospitalization were also left out: newborns, deliveries, trauma and life-threatening diseases (see Additional file 2), as were cases involving therapeutic operations that required hospitalization, insofar as they were not the consequence of an ACSC. Consequently, the following operations associated with specific ACS conditions were deemed not to be exclusion criteria: operation on stomach, peritoneum and oesophagus for bleeding ulcer; lower limb amputation for gangrene; minor operation on uterus and operation on vagina for pelvic inflammatory diseases; minor operation on mouth and teeth for dental conditions. Diagnoses and surgical SQLape® categories were used to identify these exclusion conditions [27].

### Predictors of PAH

All independent variables were measured between January 1 and June 30 2005, which corresponds to the observation period prior to the cohort zero time (1 July 2005).

The characteristics of the insured were:

–their canton of residence (seven cantons that accounted for less than 0.5% of the insured were grouped with neighbouring cantons);

–gender and age by decade;

–morbidities deduced from inpatient diagnoses (up to 10 ICD-10 codes) and outpatient dispensed drugs (Anatomical Therapeutical Chemical, ATC codes) according to the SQLape® grouper, which is suited to the nomenclatures used in Switzerland (adaptation of ICD-10 diagnostic codes and ICD-9-CM procedures codes, as well as specific pharmaceutical codes). Clinically related SQLape® categories with a similar PAH risk were grouped (see Additional file 3 for a description of these morbidity groups). Although several chronic conditions have been consistently associated with PAH risk [28,29], it is the number of chronic conditions suffered by the patient that dramatically increases their PAH risk [30]. A case mix measure, therefore, should capture the cumulative effect of multiple conditions. Because Charlson and Elixhauser indices consider only a limited number of chronic conditions [31], we systematically extended the analysis to all groups of acute and chronic diseases (Additional file 3). A morbidity group identified both from inpatient and outpatient information was considered only once. When a condition could be classified into several categories related to the same pathology, only the most severe was retained (severe infection > complicated infection > other infection > urinary infection; complicated diabetes > diabetes without complications). Patients who could be classified into more than half of the morbidity categories related to an ACSC were allocated a specific category, referred to as “ACSC related multimorbidity” (see Table 1).

**Table 1:** **Part A. Multivariate analysis of PAH incidence rates using different risk adjustment models** (**N** = **2**,**022**,**019**)

		**Adjustment models**									
			**Age-sex**	**Inpatient morbidity**	**Outpatient morbidity**	**Whole morbidity**			**Number of medical visits**		
***Variables***	**Number**	**(%)**	**IRR**	**IRR**	**IRR**	**IRR**	**95% CI**		**IRR**	**95% CI**	
Demographic											
Men 0-10	96,229	(4.8)	2.53	2.54	2.80	2.82	2.52	3.16	5.28	4.70	5.93
Men 11-20	110,428	(5.5)	0.98^ns^	1.02^ns^	1.18	1.21	1.06	1.39	2.71	2.36	3.11
Men 21-30	125,720	(6.2)	0.98^ns^	1.00^ns^	1.18	1.19	1.04	1.36	2.48	2.17	2.84
Men 31-40	156,812	(7.8)	1.01^ns^	1.03^ns^	1.16	1.16	1.03	1.32	2.14	1.89	2.43
Men 41-50	164,037	(8.1)	1.30	1.29	1.39	1.37	1.22	1.53	2.08	1.85	2.33
Men 51-60	137,066	(6.8)	2.31	2.15	1.97	1.85	1.68	2.09	2.33	2.09	2.60
Men 61-70	101,636	(5.0)	4.08	3.48	2.66	2.42	2.17	2.69	2.60	2.34	2.89
Men 71-80	65,069	(3.2)	7.78	6.12	3.67	3.25	2.91	3.61	3.28	2.96	3.65
Men 81-90	32,187	(1.6)	12.95	9.80	5.26	4.67	4.16	5.24	4.55	4.08	5.08
Men 91-100	6,655	(0.3)	15.52	12.86	7.79	6.87	5.72	8.27	6.65	5.62	7.88
Women 0-10	92,082	(4.6)	2.03	2.00	2.34	2.32	2.06	2.61	5.05	4.47	5.70
Women 11-20	104,850	(5.2)	1.10^ns^	1.13^ns^	1.30	1.32	1.16	1.45	2.65	2.11	3.03
Women 21-30	123,566	(6.1)	1.26	1.27	1.32	1.34	1.18	1.52	1.44	1.27	1.63
Women 41-50	157,341	(7.8)	1.16	1.13	1.09^ns^	1.08	0.96	1.22	1.55	1.37	1.74
Women 51-60	134,850	(6.7)	1.68	1.59	1.35	1.19	1.16	1.45	1.87	1.67	2.09
Women 61-70	105,225	(5.2)	2.87	2.61	1.81	1.73	1.55	1.93	2.29	2.06	2.55
Women 71-80	81,157	(4.0)	5.01	4.28	2.36	2.24	2.00	2.50	2.74	2.47	3.05
Women 81-90	53,433	(2.6)	9.36	7.57	3.91	3.66	3.28	4.08	4.17	3.75	4.63
Women 91-100	15,375	(0.8)	9.39	8.08	4.86	4.54	3.93	5.26	5.12	4.45	5.88
*ACSC related conditions*											
Bronchitis and asthma	17,302	(0.86)		3.05	3.65	3.07	2.88	3.27	2.12	2.01	2.23
Diabetes, complicated	2,627	(0.13)		1.61	-	1.19	0.99	1.42	1.03	.89	1.20
Diabetes, no complicated	14,714	(0.73)		1.14^ns^	1.89	1.75	1.62	1.90	1.33	1.24	1.42
Epilepsy	6,058	(0.30)		7.57	2.48	2.85	2.54	3.20	1.62	1.47	1.78
Female genital tract	20,589	(1.02)		1.50	1.41	1.24	1.13	1.36	0.70	0.65	0.77
Gastro-intestinal tract	34,765	(1.72)		2.33	1.41	1.37	1.30	1.46	1.06	1.01	1.11
Heart diseases	22,805	(1.13)		1.40	1.37	.99	.99	1.07	1.03	.97	1.09
HTA and other circulatory disorder	75,163	(3.72)		0.94^ns^	2.73	3.00	2.75	3.08	1.13	1.08	1.18
Infection, complicated	2,526	(0.12)		2.85	-	2.88	2.35	3.53	1.32	1.12	1.55
Infection, other	65,106	(3.22)		3.53	2.81	3.01	2.86	3.17	1.19	1.14	1.24
Infection, severe	1,699	(0.08)		3.47	-	3.85	3.14	4.71	1.91	1.63	2.24
Intestinal or urinary obstruction	3,640	(0.18)		0.97^ns^	-	.74	.60	.91	.52	.44	.63
Nutritional anemia	4,036	(0.20)		1.67	-	1.57	1.35	1.84	1.24	1.09	1.41
Severe lung disease	7,603	(0.38)		2.94	-	2.20	1.98	2.44	1.54	1.42	1.67
Urinary infection	4,347	(0.21)		1.57	-	1.87	1.48	2.38	1.07	.87	1.31
ACSC related multi-morbidity	2,281	(0.11)		17.07	15.13	29.39	25.33	34.10	5.12	4.55	5.76
*Comorbidity categories*											
Cancer	7,944	(0.39)		0.83	0.94^ns^	.70	.62	.78	.61	.55	.68
CNS diseases	3,014	(0.15)		1.22	-	1.03	.88	1.21	1.00	.88	1.14
Endocrine diseases	8,408	(0.42)		1.13^ns^	0.97^ns^	1.02	.92	1.13	.98	.90	1.06
Liver & biliary tract	6,272	(0.31)		1.12^ns^	1.04^ns^	.94	.83	1.06	.87	.79	.96
Mental disorders	51,605	(2.55)		1.51	1.52	1.48	1.40	1.57	1.05	1.01	1.10
Metabolic disorders	19,825	(0.98)		1.33	1.26	1.08	1.00	1.16	1.04	.98	1.10
Nephritis	6,841	(0.34)		1.22	-	1.05	0.94	1.16	1.16	1.06	1.26
Other lung disease	5,478	(0.27)		1.28	.76^ns^	1.06	0.95	1.19	1.12	1.03	1.23
Pain & chronic restriction of mobility	27,280	(1.35)		0.91^ns^	1.22	.93	.87	.99	.80	.76	.85
Systemic rheumatic diseases and transplantation	1,388	(0.07)		1.16^ns^	1.90	1.29	1.05	1.58	1.23	1.04	1.45
Skin diseases	35,802	(1.77)		1.26	1.45	1.32	1.24	1.40	.99	.95	1.04
Thrombosis	39,704	(1.96)		0.81	1.36	1.32	1.24	1.39	1.08	1.04	1.45
Trauma	9,074	(0.45)		0.62	-	.53	.47	.60	.49	.45	.54
*Physician self*-*dispensation*			-	-	1.16	1.15	1.11	1.20	.92	.89	.95
*2006 follow*-*up*	1,686,481	(83.41)	2.02	2.16	1.74	1.80	1.64	1.97	.99	.90	1.08
*Number of medical contacts*									*See Figure* 1		
						**Measures of model fit**					
AIC			179,024	172,473	162,835	159,196			130,962		
BIC			179,299	173,112	163,361	159,847			131,776		
Pseudo R2			5.2	8.7	14.1	15.8			30.8		

CI: confidence interval; IRR: incidence rate ratio; ns: not significant (p ≥ 0.05).

The intensity of care was measured by the number of physician visits during the six-month observation period (before July 2005), including ambulatory visits made by hospital physicians. However, we excluded ambulatory consultations with a radiologist and hospital visits in the 24 hours prior to hospitalization. The number of physician visits may depend on several factors, including disease severity, the propensity of the subject to seek care, and the physicians’ behaviour.

Several cantons allow physicians to dispense drugs to patients directly (self-dispensation). In such cases, information on dispensed drugs was missing. To adjust for a possible bias, we introduced an additional variable, which took the value of 1 if self-dispensation represented more than 5% of drug costs.

To examine the influence of cantonal differences in health care supply and demand, cantonal level variables were obtained from the SFSO [32]. A higher number of ambulatory care facilities, reflected in the annual costs for ambulatory care services per inhabitant, were expected to be associated with lower PAH rates, whereas greater hospital capacities were expected to be linked with higher rates. Health care supply was measured by the number of primary care independent office-based general practitioners (generalists, internists, and paediatricians), the number of independent office-based clinical specialists, as well as the number of hospital beds and pharmacists. All were expressed per 1,000 inhabitants.

The aim of using demand variables was to identify potential variations in the propensity to use ambulatory care services. A higher level of education (average number of years), higher annual income per capita, a higher proportion of urban residency, and a higher proportion of insured persons who use primary care providers as gatekeepers might be variables associated with a greater propensity to seek medical care earlier, and thus with lower PAH rates.

Other variables had a possible link to poor health (unemployment rate and low deductible with a ceiling effect) or to reduced access to physicians (higher deductible with a floor effect).

### Statistical analysis

The number of PAH observed per patient (count data) was modeled by a binomial negative regression with the number of follow-up days as exposure [33]. To remove bias due to unmeasured characteristics linked to the fact of changing insurer, a dummy variable was added in the model (2006 follow-up: 1 = yes, 0 = no). The demographic model included only gender and age. The clinical model accounted for morbidities in three ways: outpatient conditions inferred from data on dispensed drugs; inpatient conditions only; both conditions. All morbidity categories were included as dummy variables, and were retained if IRR differed significantly from 1.0 in at least one model. The full model included all available variables, including the number of physicians’ visits with three dummy variables: “no visit” (1 if zero visit), “20-29 visits” (1 if the number of visits is between 20 and 29) and “30 and more visits” (1 if there are at least 30 visits).

The Akaike information criterion (AIC) and the Bayesian information criterion (BIC) were used to assess the predictive performance of the models and a *χ*^2^ was used to test the goodness of fit [34]. The relative contribution of the independent variables to predicting PAH was gauged by comparing pseudo-R squared across the alternative models.

While information on inpatient diagnoses are routinely available from state agencies, data on drugs and ambulatory care use are generally available from payers’ organizations alone. To assess how the omission of additional information on health status affected the cantons’ profiling based on adjusted PAH rates, we plotted the difference (y-axis) in the expected number of PAH by canton under nested models, i.e. one based on complete information and one with fewer predictors, against the average expected values (x-axis) [35]. This method is similar to Bland and Altman plots for assessing agreement between two measurement methods, which have been shown to be more appropriate than the often misleading correlation coefficient [36]. The selection of risk adjustors was determined by the availability of data reflecting patient morbidity: inpatient diagnoses collected by the SFSO; morbidity based on information about dispensed drugs collected by insurers and billing organizations; and complete data [37]. The expected number of PAH per canton was computed by summing individual predicted counts. We determined the lower and upper limit of y-random variation according to the method adopted by Campbell et al. [38]. We also added two guidelines to the graph, each of which indicated a 10% relative increase or decrease in the standardized ratio (see Additional file 4 for computation details). Cantons lying above or below these lines were those whose standardized ratio increased (poorer performance) or decreased (better performance) by more than 10%.

The Spearman rank correlation coefficient (r_s_) was used to test if there was an association between the cantonal standardized ratio of PAH and each thematic group of cantonal variables (for α = 0.05, significant if r_s_ ≥0.472, 18 degrees of freedom). Estimates of the standardized ratio for each canton were obtained by incorporating cantonal effects as fixed in the final model.

All computations were performed using Stata version 11.

## Results

The studied population of over two million people represented 27% of the general Swiss population in 2005 and 2006. The age, gender and deductible distribution, as well as mortality and hospitalization rates were similar between the studied population and the Swiss population (Table 2). Cantonal distribution differed markedly between the two populations, reflecting the preferential coverage that the four health insurance companies offered in certain cantons.

**Table 2:** **Characteristics of the studied and Swiss populations** (**2005**–**2006 average**)

**Characteristics**	**Studied population**	**Swiss population**
	**N = 2,022,019**	**N = 7,483,934**
Age group in years, %		
0–19	18.9	21.8
20–39	27.2	27.1
40–64	35.8	35.0
65–79	12.1	11.5
80 and over	6.0	4.6
Female, %		
Deductible level, %	50.7	51.0
<300	14.6	14.1
300	45.0	45.1
>300	40.4	40.8
Death rate per 1,000 patients, per year	7.7	8.1
Incidence rate of hospitalization per 1,000 insured, per year	144	149
Incidence rate of PAH per 1,000 insured, per year	6.33	6.40

Sources.

Swiss Federal Statistical Office. Neuchâtel. Resident population by age from 1960 to 2009. [http://www.bfs.admin.ch/bfs/portal/fr/index/themen/01/02]

Hospital statistics 2005. Standard tables. Neuchâtel, SFSO, 2007.

Hospital statistics 2006. Standard tables. Neuchâtel, SFSO, 2008.

There were 23,129 ACSC hospitalizations during the follow-up period. Among them, 2,829 had a severe comorbidity justifying the hospitalization; 2,701 involved a therapeutic operation requiring an inpatient stay; and 550 satisfied both of these exclusion criteria. There were 17,049 PAH during the follow-up period, corresponding to 6.3 per 1,000 insured, per year. Observed PAH rates per canton varied from 1.4 to 9.6 per 1,000 insured, per year.

Table 1 shows the results of multivariate regressions. The relationship between age and ACSC rates was depicted by a J curve, with the lowest rate for category 31–40 among women and 11–40 among men, and a sharp increase over that age. Across most age categories the risk was higher among men than among women. Even after adjusting for morbidity, the effect of demographic variables on PAH remained, albeit to a lesser extent. With the exception of hypertension, diabetes without complications, and intestinal or urinary obstruction, all inpatient ACSC-related conditions increased PAH rates. Risks associated with those conditions were rather similar when outpatient morbidity was added. Most other SQLape categories that were not clinically related to ACSC had no effect on PAH rates. Only a few increased the risk significantly: mental disorders, thrombosis, skin diseases, systemic rheumatic diseases and transplantation, metabolic disorders. Hospitalizations for trauma presented a lower risk. The exclusion criteria applied to ACSC hospitalizations probably explains why the PAH risk was lower for cancer, pain and chronic restriction of mobility, and intestinal/urinary obstruction.

**Figure 1:**
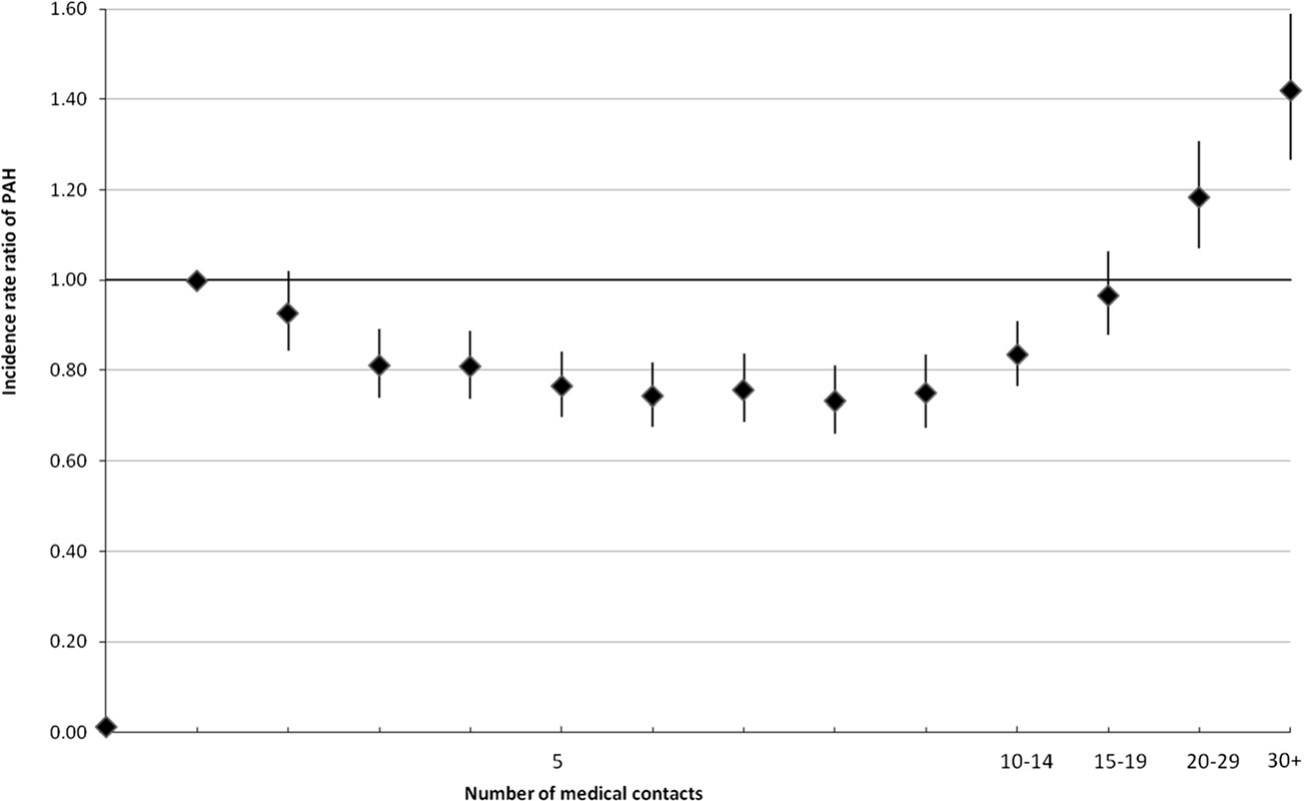
**Potentially avoidable hospitalization and frequency of medical contact.** Vertical lines: 95% CI.

Figure 1 shows that the frequency of contact with a physician prior to follow-up had a strong influence on the PAH risk. Subjects with no previous contact had a very low risk (IRR versus one contact: 0.01). Among subjects who had at least one medical consultation, the PAH risk was lower for those who had made 19 or fewer physician visits, but sharply increased once this frequency rate was exceeded. The inclusion of the frequency of contact in the model reduced the effect of morbidity categories, and even “overcompensated” for benign conditions like female genital tract disorders, whose coefficient became negative.

The goodness of fit improved as more explanatory variables related to patient health status were introduced. The pseudo R^2^ was 5.2% with demographic variables, 8.7% with inpatient illnesses, 14.1% with outpatient illnesses, and 15.8% when all detectable illnesses were taken into account. Adding the frequency of physicians’ visits dramatically improved the pseudo R^2^ (30.8%), mainly because of the considerably lower risk of PAH among numerous individuals who did not consult a doctor. AIC and BIC fell similarly. Differences between observed and predicted probabilities under the full model were negligible: Ҳ_0_ ^2^ = 0.003, Ҳ_1_ ^2^ = 4.71, Ҳ_2_ ^2^ = 17.4, Ҳ_3_ ^2^ = 4.00, Ҳ_4_ ^2^ = 0.27, indices referring to number of predicted events. Observed and expected rates of two events with the greatest differences in rates were 0.55 and 0.62 per 1,000 events.

**Figure 2:**
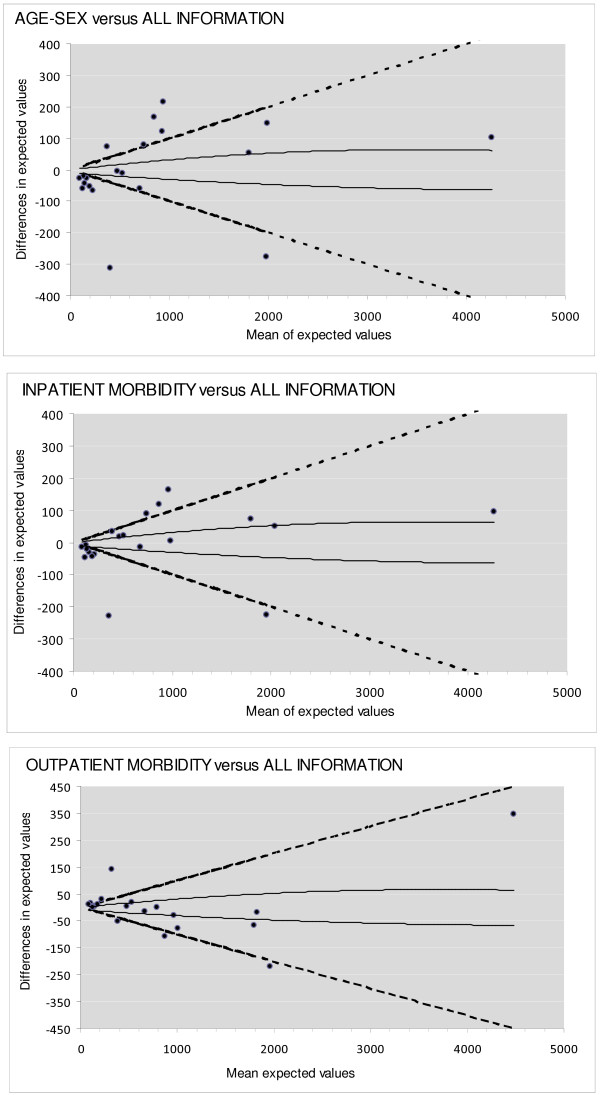
**Differences in expected values across the adjustment models.** Solid line: lower and upper limit of random variation. Dotted line: 10% increase of decrease of expected values.

Figure 2 shows the impact of different adjustment strategies on the cantons’ performance profiling. We considered that having either no contact or 20+ visits reflected the severity of the condition. However, a frequency rate of 19 or less, which was associated with a lower PAH risk, might be attributable to prevailing medical practices. Agreement between the demographic model and the full model was acceptable for less than one third of cantons.

Estimates of the standardized ratio per canton varied from 0.50 to 1.34 (Additional file 5). Four cantons were low outliers and six were high outliers.

We found no significant association between the cantonal standardized ratio and the characteristics of cantonal primary care supply or demand (Table 3).

**Table 3:** Association between cantonal supply and demand and PAH standardized ratio

	**Percentiles cut**-**off**			
	**25**	**50**	**75**	**Spearman coefficient**^**a**^
Sickness funds’ annual costs for ambulatory care services, CHF	1.996	2.251	2.726	−0.270
Pro-pharmacy proportion^b^	0.082	0.268	0.414	0.048
Urban population proportion^c^	0.557	0.742	0.941	−0.081
Pharmacy density	0.136	0.190	0.362	−0.082
General practitioners’ density	0.583	0.620	0.681	0.162
Specialist practitioners’ density	0.999	1.172	1.608	−0.187
Hospital beds density	4.637	5.518	6.055	0.212
Annual cantonal income per inhabitant, CHF	44.141	49.282	55.027	−0.067
Educational level^d^	12.686	12.839	13.028	−0.169
Deductible (CHF)	648	693	736	−0.290
Gatekeeper proportion	0.031	0.073	0.147	0.299
Unemployment rates	30.70	32.90	41.81	−0.140

Rates are expressed per 1,000 inhabitants.

^a^significant if ≥0.472 (18 degrees of freedom).

^b^proportion of medication costs dispensed by physicians.

^c^proportion of populations living in a town (>10,000 inhabitants).

^d^average number of years in education.

## Discussion

Although easily identified from the main diagnosis coding of inpatient stays, ACSC lists have varied substantially from one study to the next, without a rigorous argument to justify the inclusion and exclusion criteria [4]. In our study, 25% of cases identified by inpatient main diagnoses were removed by applying exclusion criteria which detected surgery or severe comorbidities for which hospitalization was appropriate.

Age and gender were insufficient to predict PAH risk. Taking into account all possible clinical information is of utmost importance for a fair comparison between care providers groups or regions. Inpatient diagnoses, which are available in most developed countries, should therefore be used. We recommend supplementing diagnosis-based morbidities with drug-related information (often available from insurers’ data) because of the substantial change in expected rates. Inpatient diagnoses have a limited ability to describe the disease burden because only a minority of patients is hospitalized and comorbidities like hypertension are often not recorded in hospital data. Studies have consistently found that combining pharmacy and inpatient data is an effective way of predicting care costs and utilization [39,40]. The full model provided a relatively high pseudo R^2^ value (30.8%), but further research is needed to assess predictive performances for other settings in a cross-validation procedure (prediction parameters computed on a random 50% development sample, tested on the remaining 50% validation setting).

Most ACSC-related conditions, and the few other chronic diseases associated with a PAH risk, were detectable from the data on dispensed drugs. Pharmacy-based morbidity measures, however, have limitations. Distinguishing hypertension from heart disease is problematic due to the fact that the same drug is used to treat both conditions. Consequently, heart disease which showed a higher risk in the inpatient model (accurate coding) showed a lower risk in the drug-based morbidity models (poor sensitivity). The opposite was observed for hypertension (good sensitivity of drug screening). Similarly, in the morbidity-based model diabetes with complications (only identified by diagnoses) appeared less risky than complication-free diabetes (good sensitivity of drug screening). Another limitation is that drugs information allows for the detection of only a handful of diseases [41].

Information on previous medical visits is also useful for identifying the healthiest individuals (without any visit) and the sickest (more than 19 visits within six months). Among patients having at least one consultation, PAH risk decreased with the frequency of consultations, and then rose sharply when it exceeded 19. This suggests that medical consultations in Switzerland have a preventive effect on PAH. We did not include the category “1 to 20 consultations” in the adjustment model because it might reflect medical practices rather the severity of the disease. In contrast, we retained the very high use of care category, as this has been associated with a high level of psychological distress, chronic conditions and socioeconomic disadvantage rather than with the practice behavior of physicians [5,42].

The 2006 follow-up dummy variable indicated that the risk was twice as high for people insured by the same insurer in 2006 even after adjusting for whole morbidity. However, the IRR was no more significant (0.90-1.08) after adjusting for medical visits. The most probable explanation was a higher propensity to switch to alternative health plans (restricted provider choice, level of deductible, gate keeper) among the insured who were in good health in 2005.

Risk adjustment for severity is a complex issue which requires integrating electronic data recorded during clinical care from multiple sources. These data may be difficult or expensive to obtain. Our pilot study showed the feasibility of such integration. The adoption of these data models is expected to increase rapidly in real-world settings, spurred by the financial incentives of monitoring the effectiveness of clinical care practices [43]. Some authors [44], however, cautioned against over-adjusting for patients characteristics, since primary care might also have an impact on the prevalence and the severity of illnesses through primary or secondary prevention, for instance.

After proper adjustment, the rate ratios between the lowest and highest outliers decreased from 6.7 (unadjusted IRR ratio: 9.61/1.43) to 2.7 (full model: 1.45/0.54), indicating that interregional differences were largely explained by the health status of the insured. Nevertheless, this residual variation merits further investigation. The lack of association between supply/demand factors and PAH risk in Switzerland might suggest that disparities in rates reflect differences in individual physician practices rather than varying levels of access to care. For example, physicians who were more experienced, practiced according to clinical guidelines or tended to use managed care plans, had patients who experienced fewer admissions for COPD and pneumonia [28].

Our findings contradict the correlation between PAH and access to care (in particular the supply of primary care physicians), which has been found consistently in studies of the urban American population [17,45]. However, Medicare beneficiaries living in regions with better access to primary care did not experience lower rates [46]. Although socioeconomic indices have been associated with PAH rates in many USA studies [18,47,48], confounding factors such as increased disease prevalence or severity might contribute, at least partly, to this association. Such associations are indeed weaker in settings where there is universal access to care [49-51]. Nonetheless, our regional analysis may miss relationships detected in more contrasted areas like small geographic areas or countries.

Variations between rates might also reflect factors outside the control of ambulatory care, such as the admission policies of individual hospitals [52], coding practices and unmeasured illness severity. Despite the apparent face validity of ACSC admissions, there are still many important issues from a clinical perspective that remain unanswered, such as how preventable are hospitalizations for a given condition [53].

## Conclusion

Addressing the issues of proper definition (list of exclusion criteria), risk adjustment, and the data collection burden would improve the usefulness of potentially avoidable hospitalization rates for extended application beyond a rough area level indicator of the whole ambulatory care system. PAH risk was strongly associated with patients’ clinical characteristics regardless of age and gender. A very high use of visits reflecting severe conditions dramatically increased PAH risk.

Our study underscores the importance of adjusting for many predisposing health conditions (both chronic conditions drawn from previous studies as well as other such conditions) when using PAH as an indicator of quality. The use of ambulatory care had a preventive effect on risk in Switzerland. We did not find any demand or supply effect to explain the large variability in PAH adjusted rates.

## Abbreviations

ACSC: Ambulatory care sensitive conditions; AIC: Akaike information criterion; BIC: Bayesian information criterion; ATC: Anatomical therapeutical chemical; COPD: Chronic obstructive pulmonary disease; ICD-9-CM: International Classification of Diseases, 9^th^ Revision, Clinical Modification; ICD-10: International Classification of Diseases, 10^th^ Revision; IRR: Incidence rate ratio; PAH: Potentially avoidable hospitalization; SFSO: Swiss Federal Statistical Office.

## Competing interests

Yves Eggli is the author of the SQLape patient classification system used in the article and its promoter through the SQLape s.a.r.l. His co-authors do not have any competing interests.

## Authors’ contributions

YE was in charge of the design, drafted the manuscript and supervised the preparation of data, statistical analysis and analysis of results. BD computed most statistical analyses. ES prepared data and made first data analyses. PH participated to the design, to the interpretation of medical results, made supplementary statistical analyses and co-drafted the manuscript. All authors read and approved the final manuscript.

## Pre-publication history

The pre-publication history for this paper can be accessed here:

http://www.biomedcentral.com/1472-6963/14/25/prepub

## Supplementary Material

Additional file 1 List of ICD 10 codes included in the definition of Ambulatory Care Sensitive Conditions in comparison to Purdy’s list. Click here for file

Additional file 2 Comorbidities exclusion criteria. Click here for file

Additional file 3 Content of morbidity groups used to adjust for case mix. Click here for file

Additional file 4 **Computation of guidelines of Figure **2**.** Click here for file

Additional file 5 Observed rates of PAH by canton and canton specific incidence rate ratio using different adjustment models. Click here for file
