# Supplementary material for: Comparing potentially avoidable hospitalization rates related to ambulatory care sensitive conditions in Switzerland: the need to refine the definition of health conditions and to adjust for population health status
Source: BMC Health Serv Res. 2014 Jan 20;14:25. doi: 10.1186/1472-6963-14-25 (PMC3902189; doi:10.1186/1472-6963-14-25)
Supplement: Additional file 1 — List of ICD 10 codes included in the definition of Ambulatory Care Sensitive Conditions in comparison to Purdy’s list. [file 1472-6963-14-25-S1.doc]

Additional file 1. List of ICD 10 codes included in the definition of Ambulatory Care Sensitive Conditions in comparison to Purdy’s list

| *ACSCs* | *ICD-10 codes listed by Purdy et al.* | *Additional codes* | *Suppressed codes from Purdy’s list* | *Potentially avoidable hospitalisations* |
| --- | --- | --- | --- | --- |
| Angina | I20, I240, I248, I249, I250, R072, R073, R074, Z034, Z035 |  |  | 15.28 % |
| Asthma | J450, J451, J458, J459, J46 |  |  | 3.40 % |
| Cellulitis | I891, L01, L02, L03, L04, L080, L088, L089, L88, L980 | J340 |  | 5.79 % |
| Congestive heart failure | I11, I130, I255, I50, J81 | I132 |  | 8.68 % |
| Convulsions and epilepsy | G253, G40, G41, R560, R568 |  | O150, O151, O152, O159 | 5.63 % |
| Chronic obstructive pulmonary disease | J20, J40, J41, J42, J43, J44, J47 |  |  | 12.79 % |
| Dehydratation and gastroenteritis | A020, A04, A059, A072, A080, A081, A083, A084, A085, A09, K52 | A050, A052, A053, A054, A058, A082 | E86 | 9.96 % |
| Dental conditions | A690, K046, K047, K121, K122, K131 |  | K02, K03, K04, K05, K06, K08, K098, K099, K12, K13 | 0.52 % |
| Diabetes complications* | E10.0-E10.1, E11.0-E11.1, E12.0-E12.1, E13.0-E13.1, E14.0-E14.1 |  | E10.2-E10.9, E11.2-E11.9, E12.2-E12.9, E13.2-E13.9, E14.2-E14.9 | 1.04 % |
| Ear, nose and throat infections | H66.0, H66.1, H66.2, H66.3, H66.4, J02, J03, J040, J06, J312 | H600, H601, H602 | H66.9, H67 | 3.33 % |
| Gangrene | R02 |  |  | 0.15 % |
| Hypertension | I10, I1191 |  |  | 3.19 % |
| Influenza and pneumonia** | A481, A70, J13, J14, J153, J154, J157, J159, J160, J168, J181, J182, J188, J189 |  | J10,J11,J12 | 12.58 % |
| Iron or other nutritional deficiency anaemia | D500, D508, D509, D510, D511, D512, D513, D518, D520, D521, D528, D529 | D519 | D531, D571, D580, D581, D590, D591, D592, D599, D601, D608, D609, D610, D640, D641, D642, D643, D644, D648 | 2.18 % |
| Nutritional deficiency | E40, E41, E42, E43, E550, E643 |  |  | 0.00 % |
| Other vaccine preventable diseases | A35, A36, A37, A80, B05, B06, B161, B169, B180, B181, B26, G000, M014 | J10 (>65 years old) |  | 0.15 % |
| Pelvic inflammatory disease | N70, N73, N74 |  |  | 1.21 % |
| Perforated/bleeding ulcer | K226, K250, K251, K252, K254, K255, K256, K260, K261, K262, K264, K265, K266, K270, K271, K272, K274, K275, K276, K280, K281, K282, K284, K285, K286, K920, K921, K922 |  | K20, K210, K219, K221 | 5.06 % |
| Pyelonephritis | N10, N11, N12, N136, N159 |  | N300, N308, N309, N390 | 9.07 % |

*The revised definition includes only diabetic acute complications (hyperosmolar, ketoacidosis and other diabetic coma)

** Influenza due to influenza virus had been included in other vaccine preventable diseases
