# Supplementary material for: Comparing potentially avoidable hospitalization rates related to ambulatory care sensitive conditions in Switzerland: the need to refine the definition of health conditions and to adjust for population health status
Source: BMC Health Serv Res. 2014 Jan 20;14:25. doi: 10.1186/1472-6963-14-25 (PMC3902189; doi:10.1186/1472-6963-14-25)
Supplement: Additional file 2 — Comorbidities exclusion criteria. [file 1472-6963-14-25-S2.doc]

Additional file 2. Co-morbidities exclusion criteria

Obstetrics

- Newborns

- Threatened premature labor

- Abortion

- Delivery

Trauma

- Major intracranial injury

- Other severe injury

- Vertebral column injury

- Skull injury

- Fracture of pelvis

- Chest injury

- Eye injury

- Third degree or extended burns

- Major intracranial injury

- Injury of kidney or non specified internal organ

Life threatening diseases

- Disseminated intravascular coagulopathy

- Collapse (except for dehydration)

- Acute myocardial infarction

- Pulmonary embolism

- Ruptured aneurysm of large vessel

- Transplant rejection

- End stage renal disease

- Non traumatic cerebral hemorrhage

- Meningitis or encephalitis

- Cerebrovascular accident

- Occlusion of cerebral or precerebral artery

- Hydrocephalus

Occlusions

- Intestinal obstruction

- Urinary tract obstruction

Poor health or immunity

- Cachexia

- Agranulocytosis

- Acquired immunodeficiency syndrome

Severe psychiatric disorders

- Chronic substance abuse

- Psychosis and delirium

- Nervosa anorexia

Other severe diseases

- Musculoskeletal infection

- Acute pancreatitis

- Liver abscess

- Inflammatory entero-colitis

- Guillain-Barre syndrome

- Extended bullous dermatosis
