# Supplementary material for: Comparing potentially avoidable hospitalization rates related to ambulatory care sensitive conditions in Switzerland: the need to refine the definition of health conditions and to adjust for population health status
Source: BMC Health Serv Res. 2014 Jan 20;14:25. doi: 10.1186/1472-6963-14-25 (PMC3902189; doi:10.1186/1472-6963-14-25)
Supplement: Additional file 3 — Content of morbidity groups used to adjust for case mix. [file 1472-6963-14-25-S3.doc]

Additional file 3. Content of morbidity groups used to adjust for case mix

Morbidity groups SQLape® categories

Bronchitis and asthma Chronic bronchitis and asthma

Cancer Malignant neoplasm of intestine, Malignant neoplasm of oesophagus, Malignant neoplasm of anus or rectum, Malignant neoplasm of stomach, Malignant neoplasm of the pancreas, Malignant neoplasm of brain, Neoplasm of nerve, Malignant neoplasm of upper respiratory tract, Acute lymphoid leukemia, Acute myeloid leukemia, Lymphoma, other leukemia or hematopoetic malignant neoplasm, Malignant neoplasm of urinary tract, Chemotherapy, Radiotherapy, other malignant neoplasm (this group is not allocated in presence of cancer with metastasis). Secondary malignant neoplasm, bone, Secondary malignant neoplasm, except bone

Central nervous system diseases* Cerebral ischemia, Disease of the spinal cord

Diabetes with complication* Diabetes with complication (includes acute and chronic complications)

Diabetes without complication Diabetes without complication (this group is not allocated in presence of Diabetes with complication)

Endocrine diseases Endocrine diseases

Epilepsy Epilepsy

Female genital tract Benign neoplasm of female organ, other infection of female organ, Malignant neoplasm of genital organs, Malignant neoplasm of the breast, Malignant neoplasm of the ovary, Abortion, other female disease

Gastro-intestinal tract Gastrointestinal haemorrhage, Inflammatory entero-colitis, Gastro enteritis or gastrointestinal ulcer, other disease of esophagus

Heart diseases Severe hypertension, Heart failure, Coronary arteries disorder, other acute ischemic heart disease, Gangrene, Cardiac dysrhythmia, other cardiac diseases, other disease of large vessels, other disease of vessels

HTA and circulatory disorders Aneurism of large vessel, shock, Non severe hypertension, other circulatory disorder

Infections, complicated * Heart inflammation, Peritonitis, Infection of the ovary and adnexa, Meningitis or encephalitis, Septicemia (this group is not allocated in presence of severe infection)

Infection, severe* Severe infection

Infections, others Gastro intestinal specified infection, Musculoskeletal infection, other upper respiratory infection, Acquired immunodeficency syndrome, Infection of unspecified site, Skin abscess, Infection not classified elsewhere (this group is not allocated in presence of severe or complicated infection)

Intestinal or urinal occlusion* Intestinal obstruction, Urinary tract obstruction

Liver and biliary tract Liver abscess, Liver cirrhosis, Chronic pancreatitis, other biliary disorder, Cholecystitis or angiocholitis, Hepatitis, Acute pancreatitis, Malignant neoplasm of the liver, Biliary obstruction without complication, other liver disease

Mental disorders Depression, Acute substance abuse, other psychiatric disorder

Metabolic diseases Gout and secondary arthritis, Metabolic disease or general symptoms, Metabolic or blood disorder

Nephritis* Chronic nephritis, Acute nephritis

Nutritional anaemia* Nutritional anaemia

Other lung disease Malignant neoplasm of lung, other respiratory disorder, other pulmonary disease, other operation on trachea

Pain and chronic Degenerative disease of knee, Degenerative disease of other bones, Degenerative disease of vertebra,
restriction of mobility Degenerative disease of brain, Extended paralysis, Cerebral disorder, Cachexia, Decubitus ulcer, Skin ulcer, End
 stage renal disease, Rehabilitation or palliative care

Rheumatism and transplant Severe musculoskeletal inflammation, Transplant rejection

Severe lung diseases * Interstitial pulmonary disease, Chronic respiratory failure, Acute respiratory failure, other pulmonary infection, Influenza or acute bronchitis

Skin diseases Third degree or extended burns, Extended bullous dermatitis, Localized skin infection, Inflammatory dermatitis, Malignant neoplasm of skin, Superficial injury, Open wound and non superficial burns, Obesity, Other skin disease

Thrombosis Acute myocardial infarction, other thrombosis or embolism, Thrombosis or embolism of limb, Pulmonary embolism,

Trauma* Arm and forearm injury, Fracture of pelvis, Leg injury, other muskuloskeletal injury, Skull injury, Vertebral column injury, other severe injury, Major intracranial injury, Minor cerebral injury, Chest injury, Eye injury, Injury of kidney or non-specified internal organ

Urinary infection* Urinary infection (this group is not allocated in presence of severe, complicated infection or other infections)

*Morbidity groups not allocated through drugs prescriptions (only inpatients’ diagnoses)
