# Supplementary material for: Comparing potentially avoidable hospitalization rates related to ambulatory care sensitive conditions in Switzerland: the need to refine the definition of health conditions and to adjust for population health status
Source: BMC Health Serv Res. 2014 Jan 20;14:25. doi: 10.1186/1472-6963-14-25 (PMC3902189; doi:10.1186/1472-6963-14-25)
Supplement: Additional file 4 — Computation of guidelines of Figure 2. [file 1472-6963-14-25-S4.doc]

Additional file 4**. Computation of guidelines of Figure 2**

**a) Random variation of differences of expected values (curves)**

Assuming that E_i_ follow a Poisson distribution (without overdispersion), their variance is approximately $\overline{E}$ and thus :

Var(E-E’)=var(E)+var(E’)-2 cov(E,E’)

Under the hypothesis of a high correlation (for instance 0.90) between E_i_ and E’_i_ we have:

Var(E-E’) = var(E) + var(E’) – 2 * 0.90$\sqrt{\mathrm{var}(Ei)}$ $\sqrt{\mathrm{var}(E'i)}$

= $\overline{E}$ + $\overline{E}$ - 1.8 $\overline{E}$ = 0.2 $\overline{E}$

Two standard deviations = $\pm2\sqrt{0.2\overline{E}}=0.89\sqrt{\overline{E}}$

For simplicity, we retain: $\pm\sqrt{\overline{E}}.$

**b) Magnitude of differences of expected values (lines)**

Let E_i_  the expected numbers of events for canton i omitting variables X and E’_I_ the expected number of events for canton I including variables X. Comparing adjusted rates means measuring the rate difference Δ :

$$\Delta=\frac{O}{E}-\frac{O}{E'}=\frac{O(E-E')}{E E'}\cong\frac{O(E-E')}{E'\overline{E}}$$

If we accept a maximal rate difference of 10%, we have $E'-E=10\%\overline{E}$ and therefore:

$$\Delta\max=10\%\frac{O}{E'}$$

Thus the lower and upper limits (rate difference) are easily plotted on y-axis and E’ on x-axis.
