# Supplementary material for: Comparing potentially avoidable hospitalization rates related to ambulatory care sensitive conditions in Switzerland: the need to refine the definition of health conditions and to adjust for population health status
Source: BMC Health Serv Res. 2014 Jan 20;14:25. doi: 10.1186/1472-6963-14-25 (PMC3902189; doi:10.1186/1472-6963-14-25)
Supplement: Additional file 5 — Observed rates of PAH by canton and canton specific incidence rate ratio using different adjustment models. [file 1472-6963-14-25-S5.doc]

Additional file 5. Observed rates of PAH by canton and canton specific incidence rate ratio using different adjustment models

| **Canton** | **Sample size** | **Observed rate per 1000 insured-year** | **Incidence rate ratio according the adjustment models** | | | |
| --- | --- | --- | --- | --- | --- | --- |
|  |  |  | **demographic** | **Inpatient illnesses** | **Drug based morbidity** | **All information** |
| A | 430,262 | 6.94 | 1.04 | 1.05* | 0.89* | 0.96 |
| B | 52,474 | 7.64 | 1.28* | 1.28* | 1.17* | 1.14* |
| **C** | **35,202** | **8.88** | **1.44*** | **1.37*** | **1.32*** | **1.06** |
| **D** | **96,591** | **7.48** | **1.40*** | **1.35*** | **1.27*** | **1.05** |
| E | 221,203 | 4.03 | 0.74- | 0.73- | 0.72- | 0.65- |
| **F** | **25,713** | **5.50** | **1.04** | 1.04 | **1.15** | **1.31*** |
| G | 20,984 | 2.52 | 0.52- | 0.52- | 0.66- | 0.73- |
| H | 34,435 | 4.87 | 0.92 | 0.96 | 0.96 | 1.08 |
| I | 77,647 | 1.43 | 0.26- | 0.28- | 0.32- | 0.50- |
| J | 65,494 | 7.89 | 1.35* | 1.40* | 1.24* | 1.24* |
| K | 15,911 | 6.65 | 1.11 | 1.14 | 1.13 | 1.15 |
| **L** | **77,515** | **8.22** | **1.35*** | 1.32* | **1.14*** | **1.08** |
| **M** | **28,422** | **4.30** | **0.77-** | 0.76- | **0.82-** | **0.89** |
| N | 28,229 | 3.81 | 0.89 | 0.94 | 1.04 | 1.06 |
| O | 105,728 | 9.61 | 1.73* | 1.64* | 1.50***** | 1.34* |
| P | 253,201 | 7.12 | 1.32* | 1.27* | 1.36***** | 1.12* |
| Q | 96,727 | 8.52 | 1.46* | 1.39* | 1.32***** | 1.15* |
| R | 12,518 | 4.93 | 0.91 | 0.97 | 0.96 | 1.06 |
| S | 255,741 | 4.88 | 0.87- | 0.87- | 0.91**-** | 0.90- |
| T | 88,022 | 5.81 | 0.94 | 0.94 | 0.94 | 1.01 |
| Overall | 2,022,019 | 6.33 |  |  |  |  |

The reference category is the overall risk; high and low outliers at p<=0.05 are indicated by * and - ; cantons changing their outliers status when switching from demographic to all information based model are indicated in bold.
